# A Balanced Comparison of Object Invariances in Monkey IT Neurons

**DOI:** 10.1523/ENEURO.0333-16.2017

**Published:** 2017-04-13

**Authors:** N. Apurva Ratan Murty, Sripati P. Arun

**Affiliations:** Centre for Neuroscience, Indian Institute of Science, Bangalore, India

**Keywords:** inferotemporal, invariance, object recognition, pattern recognition

## Abstract

Our ability to recognize objects across variations in size, position, or rotation is based on invariant object representations in higher visual cortex. However, we know little about how these invariances are related. Are some invariances harder than others? Do some invariances arise faster than others? These comparisons can be made only upon equating image changes across transformations. Here, we targeted invariant neural representations in the monkey inferotemporal (IT) cortex using object images with balanced changes in size, position, and rotation. Across the recorded population, IT neurons generalized across size and position both stronger and faster than to rotations in the image plane as well as in depth. We obtained a similar ordering of invariances in deep neural networks but not in low-level visual representations. Thus, invariant neural representations dynamically evolve in a temporal order reflective of their underlying computational complexity.

## Significance Statement

We effortlessly recognize objects despite changes in their position, size, and viewpoint, but the relationship between these invariances is poorly understood. Here, we compared the magnitude and dynamics of object invariances in monkey inferotemporal (IT) cortex, an area critical for object recognition. We find that invariances developed in a fixed temporal order across the visual response: size and position invariance developed first, followed by rotation and viewpoint invariance. This hierarchy of invariances was not a trivial outcome of low-level visual representations but rather reflected their computational complexity as evidenced by an analogous ordering of invariances in deep neural networks.

## Introduction

Object recognition is challenging, in part because the images of an object can vary widely across viewing conditions. Our ability to recognize objects across variations in viewing conditions is based on invariant or tolerant object representations in the higher visual areas (Connor et al., 2007; DiCarlo et al., 2012). These neural representations have been best studied in the monkey inferotemporal (IT) cortex, an area critical for object recognition, whose neurons are invariant to size, position, and viewpoint (Perrett et al., 1991; Ito et al., 1995; Wallis and Rolls, 1997; Hung et al., 2005; Zoccolan et al., 2007; Li et al., 2009; Ratan Murty and Arun, 2015). While these invariances themselves are well established, we know little about how they are related. For instance, are some invariances harder than others? Are some invariances solved faster than others in the brain? A previous study has compared size and position invariance dynamics in human MEG but without equating size and position changes (Isik et al., 2014). Such comparisons are uninterpretable because if position changes were smaller than size changes, neural responses would also change less for position than size, leading to larger position invariance. Conversely, if position changes were larger than size, it would lead to the opposite result. Thus, it is critical to compare invariances after equating image changes across transformations.

**Figure 1.**
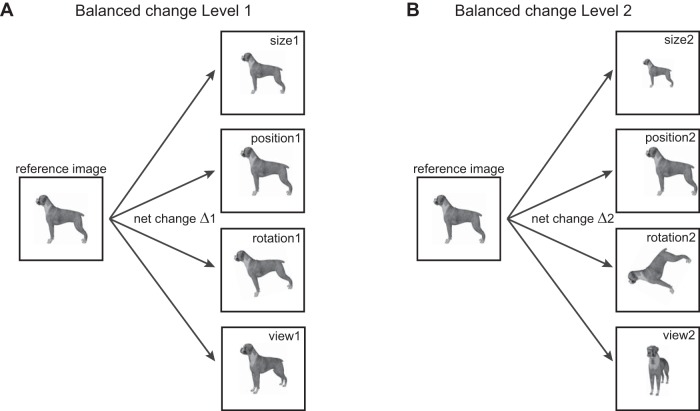
Schematic of experiment design. To compare invariances in the brain, we designed stimuli in which the net change in the image was identical across a number of identity-preserving transformations. ***A***, For balanced change level 1, we transformed the reference image in either size, position, in-plane rotation or view (i.e., in-depth rotation), with the constraint that the net change in the image is equal (depicted by Δ1). ***B***, Same as ***A*** but for change level 2.

Our goal was to perform a balanced comparison of object invariances in IT neurons. We selected four basic identity-preserving transformations: size, position, in-plane rotation (denoted as rotation) and in-depth rotations (denoted as view). For each change level, we equated the net change in pixel intensity across size, position, rotation, and view (Fig. 1). We recorded the responses of IT neurons to these stimuli to compare the magnitude and dynamics of invariant object representations. Our main finding is that there are systematic differences in the dynamics of invariance, size invariance developed early in the neural response, followed by position, and then by rotation and view invariance. By comparing with computational models of early and late visual areas, we show that these differences are not a trivial consequence of low-level representations but reflect their computational complexity.

## Materials and Methods

All animal experiments were performed according to a protocol approved by the Institutional Animal Ethics Committee of the Indian Institute of Science and the Committee for the Purpose of Control and Supervision of Experiments of Animals, Government of India. Most experimental methods and procedures have been previously described in other studies performed using the same animals (Ratan Murty and Arun, 2017). Therefore, only the details specific to this study are described below.

### Neurophysiology

We recorded from 127 visual neurons from the left IT cortex of two monkeys (*Macaca radiata*, male, aged 7 years; 83 neurons from monkey *Ka* and 44 neurons from monkey *Sa*). Results were qualitatively similar in each monkey. Recording locations were verified to be in the anterior ventral IT cortex. Recordings were performed using a 24-channel multicontact electrode (Plexon Uprobe, Plexon) using standard neurophysiological methods.

### Behavioral task

Animals were trained to perform a fixation task. On each trial, a fixation dot appeared on which the animal was required to fixate. On attaining fixation a series of eight stimuli were presented for 200 ms each and an equal inter-stimulus interval, and the animal was rewarded with juice for successfully maintaining its gaze within a window measuring 3° centered on the fixation dot. Although the fixation window was relatively large, *post hoc* analyses showed that the animals’ eye position remained closely centered throughout the trial (standard deviation of eye position during a trial, averaged across all sessions: 0.27° around the horizontal, 0.38° around the vertical).

### Stimuli and trial design

We used 10 naturalistic objects. These included four animals (camel, cow, deer, and dog) and six inanimate objects (four cars, a motorbike, and a shoe). All objects were rendered using 3D modeling software (Autodesk 3DS Max 2012, Academic License) and were presented against a black background (all 10 objects are displayed across the figures). For each object, we defined the view at which it was most elongated as the reference. The reference views of all objects were and scaled to have a longer dimension of 5.5°. We rotated this reference view in depth by two levels: 30° and 75°. To equate the different invariances, we calculated for each object the summed absolute difference across pixel intensities between the reference image and view 1 (say Δ1) and between the reference image and view 2 (say Δ2). To create the equivalent position-shifted images, we shifted the reference image until the net image change exactly matched the two change levels Δ1 and Δ2. This procedure was repeated for size and rotation. This procedure is depicted schematically in Figure 1. This ensured that the net image change is exactly the same for changes in size, position, rotation, or view. Because each object transforms differently with viewpoint, the exact variation in size/position/rotation that produced an equivalent net image change was different for each object (average scaling in size across objects: 0.87 ± 0.02× and 0.71 ± 0.02× for level-1 and level-2 changes, respectively; position change: 0.9 ± 0.1° and was 2.7 ± 0.2° contralateral relative to fixation; rotation change: 11.1° ± 1.1° and 39.6° ± 5.6°).

### Trial design

Each trial consisted of 8 stimuli presented in pseudo-random order with the constraint that two versions of the same object never occurred consecutively in a trial. Each stimulus was presented a total of eight times across trials.

### Invariant object decoding analyses

We performed a population decoding analysis to measure the overall invariance of the neural population (Hung et al., 2005). To this end, we represented the response of the entire population as a multidimensional vector with each entry representing the activity of one neuron. We then trained a multi-class linear classifier (classify function, MATLAB) to decode object identity using response vectors in each trial across the reference image of all objects and tested this classifier on trials corresponding to the transformed object images (and vice versa). The decoding accuracy reported is the decoding accuracy obtained on test data on which the classifier was never trained. This approach yields an upper bound for the information conveyed by the entire neural population were it to be recorded simultaneously.

### Computational models

We tested several model representations as detailed in the text. These are elaborated below. In each model, the response to a given image is taken as a vector of activity across all model units. The first model is the pixel model, where model units are simply pixels and unit activation is simply the pixel gun intensity (0-255). The second model is a standard V1 model comprising Gabor filters at various spatial frequencies and orientations, with input and output normalization (Pinto et al., 2008; Ratan Murty and Arun, 2015). The output of the model was the activity of 1080000 model V1 units which were used as the feature vector. The third model was a pretrained state-of-the-art deep neural network implementation (VGG-16, http://www.vlfeat.org/matconvnet/pretrained/) with 3 × 3 convolutional layers and 16 weight layers (Simonyan and Zisserman, 2015). Note that the network was not specifically trained on our stimuli. For each layer, we calculated the pairwise dissimilarities across the reference objects across units (as one minus the correlation between unit activations) and calculated the match with the pairwise dissimilarities observed in IT neurons. The conv-5 layer showed the highest match (*r* = 0.78, *p* < 0.00005) between the deep neural network and IT and therefore was used for further investigation. Subsequent layers including the fully connected layer yielded qualitatively similar results (i.e., view and rotation invariance were weaker than size and position invariance).

### Reliability of the IT representation

To estimate the reliability of the IT representation, we calculated the average correlation between the dissimilarities obtained from two independent halves of the recorded neurons (across many splits). This average correlation underestimates the true reliability, since it is calculated across two halves rather than the entire data. We therefore took as the true reliability of the IT data the Spearman-Brown corrected correlation which adjusts for this underestimate. The corrected correlation is given by rc = 2r/(r + 1), where r is the split-half correlation.

## Results

We recorded the responses of 127 visual neurons from the left IT cortex of two monkeys during a fixation task. The stimuli consisted of images of naturalistic objects whose reference images were modified across four identity-preserving transformations: size, position, rotation, and view (Fig. 1). Each transformation had two levels of change. For each level, the net pixel change (i.e., the net absolute difference in pixel intensities) in the image was equated across all transformations. This allowed us to perform a balanced comparison of invariances in IT neurons.

### Do IT neurons maintain their selectivity across transformations?

**Figure 2.**
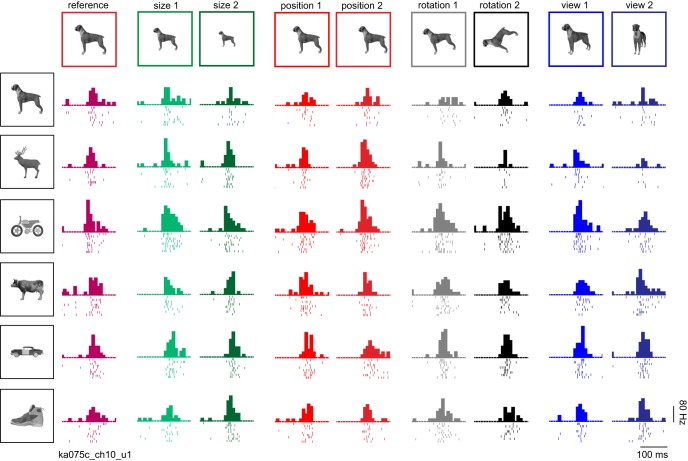
Example IT neuron. Responses of an example IT neuron for a subset of six objects across all transformations (levels 1 and 2 for size, position, rotation, and view). In each subplot, the starting point is the image onset time, each row is a single trial and ticks indicate spike times. The histogram above the raster represents the firing rate in 20-ms bins.

**Figure 3.**
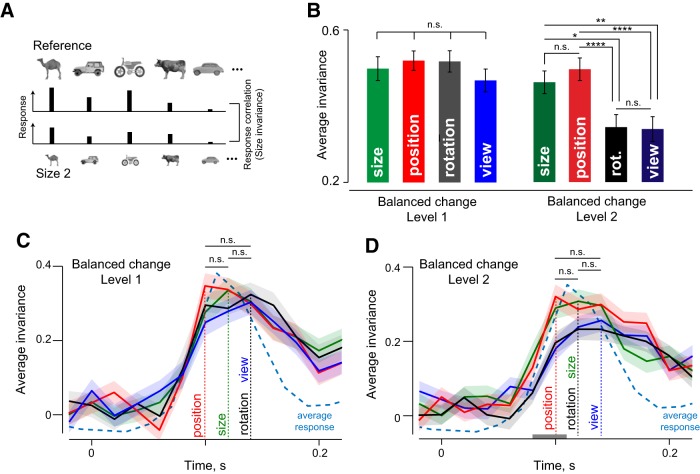
Dynamics of invariance in IT neurons. ***A***, To quantify size invariance, we calculated the correlation between the responses to the reference images of all objects (top row) and the responses to size-transformed images of these objects (bottom row). ***B***, Average size, position, rotation, and view invariance across all neurons for balanced change level 1 (left) and level 2 (right). Error bars indicate SEM. Asterisks indicate statistical significance (* is *p* < 0.05, ** is *p* < 0.005, *** is *p* < 0.0005, **** is *p* < 0.00005). ***C***, Average size, position, rotation, and view invariances (for change level 1) in 20-ms bins during the image presentation period (200 ms). Vertical dashed lines indicate the peak latency for each invariance type. The average normalized neural response (across all cells and stimuli) is displayed (cyan dashed line) for comparison. ***D***, Same as ***C*** but for larger changes (level 2). The gray bar near the *x*-axis represents the time period during which the average of size and position invariance was significantly larger than the average of rotation and view invariance (*p* < 0.05, rank-sum test across invariances of 127 neurons in each time bin).

The responses of an example IT neuron are shown in Figure 2 for a subset of the objects tested. Although the response is modulated to some degree by the identity-preserving transformations, the neuron tended to maintain its preference for the same objects. To quantify invariance, we calculated for each neuron the correlation between its responses (i.e., firing rate in a 50- to 200-ms window after image onset) to the reference and transformed images of all objects (Fig. 3*A*). Across neurons, small image changes (level 1) resulted in strong invariance in all cases, and the magnitude of invariance did not differ significantly (Fig. 3*B*; *p* > 0.05 for all pairwise comparisons, sign-rank test on invariances across neurons). Roughly half of all recorded neurons showed statistically significant (*p* < 0.05) correlations in their responses to the reference and transformed stimuli (average significant correlations: 0.81, 0.78, 0.77, and 0.77 for size, position, size, and viewpoint, respectively, across 51, 54, 63, and 51 neurons).

For large image changes (level 2), invariance did not reduce for position and size (*p* > 0.05, sign-rank test comparing level 1 and 2). Close to half of all neurons (42%) showed significant tuning correlation (average significant correlation: 0.78, 0.79 across 51 and 57 neurons, respectively, for size and position invariance). Importantly, invariance was weaker for rotation and view compared with size and position (sign-rank test comparing invariances across neurons; Fig. 3*B*). The weaker invariance was also evident in the smaller fraction (28%) of neurons with a significant tuning correlation for rotation and view (average significant correlation: 0.76 and 0.69 across 38 and 35 neurons, respectively). This fraction was significantly smaller than the fraction of neurons invariant for size and position (*p* < 0.00005, χ^2^ test). We conclude that rotation and view invariance are weaker in magnitude compared with size and position invariance.

### Dynamics of tuning correlation

To examine how these invariances develop over time in the neural response, we calculated the tuning correlation in 20-ms time bins during the image presentation period. For small image changes (level 1), all four invariances were equally strong in magnitude, and attained their peak nearly simultaneously (Fig. 3*C*). For larger image changes (level 2), there were differences in how invariances developed over time: size and position invariance were stronger in magnitude (during 80-110 ms) compared with rotation and view invariance (Fig. 3*D*, gray bar; *p* < 0.05 on rank-sum test comparing invariances across neurons for each time bin). This trend was also reflected in the peak latencies of these signals, although the differences did not attain significance (Fig. 3*D*; *p* > 0.05, sign-rank test on invariances across neurons). We conclude that size and position invariance emerge earlier compared with rotation and view invariance in IT neurons.

### Dynamics of invariant object decoding

**Figure 4.**
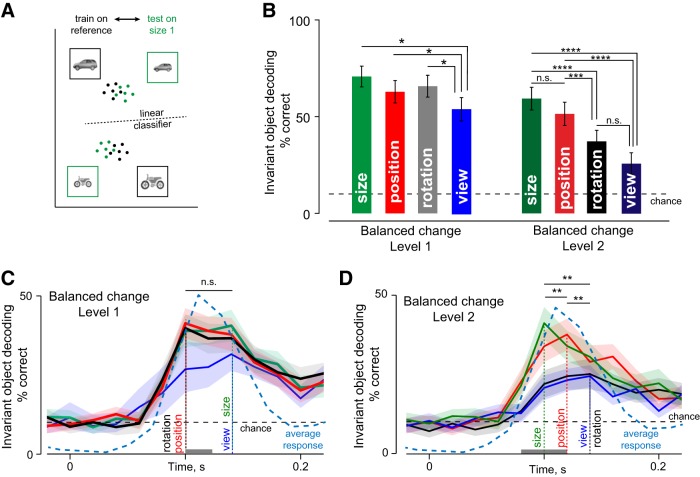
Dynamics of invariant population decoding in IT. ***A***, To quantify invariant decoding at the level of the neural population, we trained linear classifiers to identify objects in their reference images, and asked whether the same classifiers would generalize to transformed object images (in this case, to size 1). High decoding accuracy implies strong size invariance. ***B***, Invariant object decoding accuracy for size, position, rotation, and view for change level 1 (left) and change level 2 (right). Error bars indicate the SEM accuracy across trials. Asterisks indicate statistical significance as before. ***C***, Invariant object decoding accuracy for level 1 changes of size, position, rotation, and view calculated in 20-ms time bins throughout the visual response period. Thick lines indicate mean decoding accuracy, and shaded regions indicate the standard deviation estimated from 100 bootstrap estimates obtained by sampling neurons with replacement. The dotted line indicates chance decoding performance (10%). The gray bar near the *x*-axis represents the time bins during which view decoding was significantly smaller than size, position, and rotation decoding (*p* < 0.05, rank-sum test across trial-wise decoding accuracy for view versus others). The average normalized neural response (across all cells and stimuli) is displayed (cyan dashed line) for comparison. ***D***, Same as ***C*** but for change level 2.

In the preceding section, we characterized the average invariance across neurons, but a sophisticated population decoder might read out object identity more efficiently by relying more on informative/invariant neurons. To assess this possibility, we trained linear classifiers on trial-wise neuronal responses to the reference images of all objects and tested them on the responses to the transformed images (Fig. 4*A*; see Materials and Methods). The accuracy of the resulting classifier therefore measures the overall invariance of the neuronal population.

For the population response measured in a 50- to 200-ms window, the invariant population readout for small (level 1) changes was well above chance across size, position, rotation, and view transformations (Fig. 4*B*). However, view generalization was significantly smaller in magnitude compared with the others (Fig. 4*B*; *p* < 0.05, rank-sum test on trial-wise decoding accuracy for all pairs of invariances). For larger image changes (level 2), invariant object decoding remained significantly above chance for all transformations but was weaker for both rotation and view compared with either size or position (Fig. 4*B*; *p* < 0.005, rank-sum test on trial-wise decoding accuracy). Thus, size and position invariant decoding are stronger than rotation and view invariant decoding in IT neurons.

To assess the dynamics of invariant decoding, we repeated the above analyses on the firing rate of the neuronal population in 20-ms time bins. For small image changes (level 1), size, position, and rotation invariant object decoding had similar dynamics. However, view invariant decoding was significantly weaker in magnitude early on the response (Fig. 4*C*, gray bar; *p* < 0.05, rank-sum test on trial-wise decoding accuracy for view compared with others). These trends were reflected in the peak latencies of these signals as well (Fig. 4*C*). We assessed the statistical significance of the latency differences using bootstrap resampling: we sampled neurons repeatedly with replacement and calculated the peak latency each time. We then performed a statistical comparison using 100 bootstrap-derived peak latency estimates (the number was matched roughly to the number of cells). As an alternative measure we also calculated the fraction of estimates in which the peak latency for view invariant decoding was larger than that of position, size, or rotation. This revealed a statistically significant difference in latency for view compared with position and rotation invariant decoding (Fig. 4*C*; *p* < 0.00005, sign-rank test; view decoding latency was later than position and rotation decoding latency 84% and 81% of the estimates). However, view decoding latency did not differ from that of size invariant decoding (Fig. 4*C*; *p* > 0.05, sign-rank test, view > size latency 34% of the time).

For large image changes (level 2), the magnitude and dynamics of different invariance showed clearer differences. View and rotation invariant decoding attained a peak significantly later compared with size and position (Fig. 4*D*; *p* < 0.00005, sign-rank test across 100 bootstrap-derived estimates comparing view/rotation with size or position; view latency > size latency in 96% of estimates, view > position latency in 84% of estimates, rotation > size latency in 97% of estimates, and rotation > position latency in 78% of the estimates). View invariant decoding accuracy was also significantly weaker than both position and size decoding (*p* < 0.05, rank-sum test on trial-wise accuracy from 50-200 ms). Finally, there was no significant difference between the dynamics of view and rotation invariance (*p* > 0.05, sign-rank test on bootstrap estimates; view latency > rotation latency in 52% of the samples). Size and position invariance also showed subtle but significant differences in their dynamics: position invariance peaked later than size invariance (peak latency: 100 and 120 ms for size and position; *p* < 0.00005, sign-rank test on bootstrap samples; position > size latency in 95% of the estimates). To confirm that these differences are still present in subsets of neurons invariant to each transformation, we repeated the above analyses on the top 20/50/60 neurons with the highest tuning correlation for each transformation. This too yielded qualitatively similar results.

To sum up, the population decoding analyses reveal a clear temporal order in the magnitude and dynamics of invariance: size invariance peaks early, followed by position and then followed by rotation and view invariance.

### Do invariances covary across neurons?

**Figure 5.**
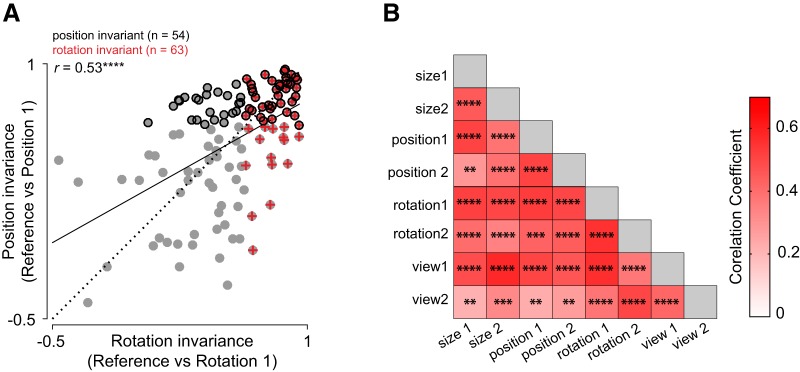
Invariances covary across neurons. ***A***, Position invariance plotted against rotation invariance across all neurons for level 1 changes. The dashed line is the unit slope line and the solid line represents the best linear fit. Position invariant neurons are marked by black circles, rotation invariant neurons are marked by red plus symbols, and all other cells are marked using gray circles. ***B***, Correlations between all pairs of invariances across neurons. Asterisks represent statistical significance as before.

The observed differences between invariances could be driven by different groups of neurons invariant to different transformations, or by the same set of neurons invariant across all transformations. We investigated this issue in two ways: First, we asked whether the strength of invariance covaries across transformations. For instance, we plotted the rotation invariance of each neuron against its position invariance. This revealed a significant correlation across all neurons (*r* = 0.53, *p* < 0.0005; Fig. 5*A*). In other words, a neuron that is strongly rotation invariant is also strongly position invariant. This was true of all pairs of invariances as well (Fig. 5*B*).

Next, we examined the degree of overlap between neurons invariant for different level-2 transformations and compared this with the overlap expected if these two properties were independently distributed. For instance, since 51 of 127 neurons (40%) show size invariance and 57 of 127 neurons (45%) show position invariance, the number of neurons expected to show both invariances assuming the two properties are independently distributed would be 23 neurons (0.4*0.45 = 18% of 127). Likewise the number of neurons expected to show size invariance but not position invariance would be 28 [0.4*(1-0.45) = 22% of 127]. In this manner, we compiled the expected number of cells in all four possible combinations (size invariance present/absent × position invariance present/absent) which yielded the expected vector of counts [23 34 28 42]. We compared this with the observed vector of counts for size and position, which was [31 26 20 50]. These two counts were significantly different from each other, as assessed using a χ^2^ test (*p* = 0.006). We conclude that size and position invariance are not independently distributed, i.e., covary across neurons. In this manner, we found that size, position, rotation, and view invariance co-occurred significantly more frequently across neurons than expected had they occurred independently (*p* < 0.05 for all pairwise comparisons, χ^2^ test; *p* = 0.072 for size versus view).

Based on the above, we conclude that the same population of neurons tend to be invariant across all identity-preserving transformations.

### Are invariant neurons clustered anatomically?

To investigate whether invariant neurons tended to occur at specific anatomic locations, we calculated the correlation between average invariance of each neuron (across all transformations) with the anterior-posterior, medial-lateral, and depth coordinates of the recorded sites (combined after subtracting the mean within each animal). This revealed no systematic correlation (*r* = −0.09, 0.07, and 0.12 for anterior-posterior, medial-lateral, and depth coordinates; all *p* > 0.1). Thus, invariant neurons show no anatomic clustering at least across the spatial scales sampled in our study.

### Does invariance for similar stimuli emerge later?

So far, we have characterized the overall invariance to a diverse set of objects. We confirmed that differences in invariance dynamics was robustly present across many random subsets of objects. However, it is possible that similar objects may show different invariance dynamics. To investigate this possibility, we repeated the invariant object decoding analyses on level-2 changes of three groups of objects: similar animate objects (camel, cow, deer, dog), similar inanimates (four cars), and dissimilar objects (car, animal, motorbike, shoe).

We obtained qualitatively similar results across the different stimuli sets. Invariant object decoding accuracy was better for size and position compared with rotation and view as before, but accuracy was higher overall for dissimilar objects compared with similar objects (average decoding accuracy for size, position, rotation, and view: 52%, 55%, 49%, and 32% for similar animates; 43%, 43%, 32%, and 29% for similar inanimates; 82%, 66%, 50%, 43% for the dissimilar objects). This is consistent with the finding that dissimilar objects show higher viewpoint invariance in IT neurons (Ratan Murty and Arun, 2015). The time course of invariant decoding also showed delayed decoding of rotation and view compared with size/position (peak latency for size, position, rotation, and view: 120, 140, 160, 180 ms for similar animates; 120, 120, 100, 140 ms for similar inanimates; 100, 100, 120, 160 ms for dissimilar objects). We conclude that size/position invariance is consistently stronger and develops earlier compared with rotation/view regardless of object structure.

### Do invariances vary in their computational difficulty?

The above results show that rotation and view invariance are harder and arise later compared with size/position invariance in IT neurons. This hierarchy of invariances could be inherited from low-level visual representations or reflect the computational complexity of achieving these invariances. To investigate this issue, we examined two low-level visual representations: a pixel model and a V1 model (see Materials and Methods). We also investigated various layers of a state-of-the-art convolutional neural network optimized for object detection (Simonyan and Zisserman, 2015). We selected the second layer of this network as a proxy for low-level representations (DNN layer 2). As a proxy for high-level representations, we selected the layer whose neural dissimilarity across the reference stimuli matched best with the IT data (conv-5 layer, correlation with IT dissimilarities: *r* = 0.78, *p* < 0.00005). We note that this high degree of agreement is consistent with recent observations that object representations in deep neural networks match extremely well with high-level visual cortex (Khaligh-Razavi and Kriegeskorte, 2014; Yamins et al., 2014) and with perception (Pramod and Arun, 2016). Importantly none of these representations were fitted to match the IT data, since our purpose was simply to compare the intrinsic nature of different invariances. We then compared these representations with the observed IT representations, as detailed below.

**Figure 6.**
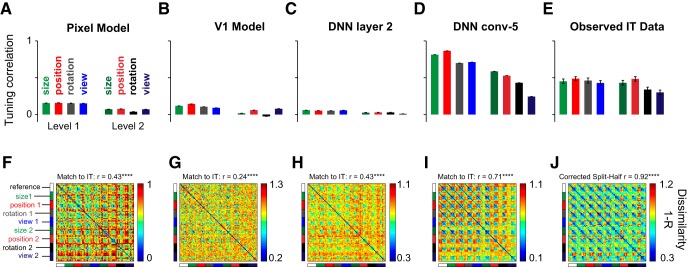
Comparison with computational models. To investigate whether the invariance differences observed in IT are a trivial consequence of low-level visual representations or reflect the underlying complexity, we compared the IT representation with several models: pixel, V1, and initial and later layers of a deep neural network i.e. DNN (see text). ***A***, Average invariance, measured as the tuning correlation between reference images and transformed images across objects for the pixel model. The bars indicate the mean tuning correlation and the error bars indicate the SEM across all units. ***B–E***, Same as ***A***, but for the V1, DNN layer 2, DNN conv-5, and IT neurons. ***F***, Pairwise dissimilarity between all pairs of 90 stimuli for the pixel model. The match to IT indicates the correlation between this matrix and the observed dissimilarities in IT (depicted in ***J***). ***G–I***, Same as ***F*** but for the V1, DNN layer 2, and DNN conv-5 layer, respectively. ***J***, Same as ***F*** but for IT neurons. The correlation above the color map represents the reliability of the IT data, which is an estimate of the upper bound achievable for any model of IT (see Materials and Methods).

We took individual units in each model and calculated the invariance exactly in the same way as for IT neurons: for each transformation we calculated the correlation between the activation of the unit for the reference and transformed images across objects. For the pixel, V1 and DNN layer-2 representations, the average tuning correlation was small as expected since images change drastically across size/position/rotation/view changes (Fig. 6*A–C*). As expected, the invariance was smaller for level-2 transformations compared with level-1 transformations. However, the average invariance (across ∼100,000 units) was relatively larger for the DNN conv-5 layer, with size and position invariance being stronger than rotation and view (Fig. 6*D*), which is similar to the pattern observed in IT (Fig. 6*E*). We note that the average tuning correlation is higher in the DNN conv-5 layer than in IT. However, this could reflect the vast difference in sampling of the two networks: in IT we have sampled only ∼100 neurons whereas for the DNN, we have sampled all 100,000 units.

Finally, we compared the overall representation in each model by calculating the pairwise dissimilarity between unit activations in each model across all pairs of images (for each pair, this is 1, correlation between the activity elicited by the two images across all units). In this representation, if the reference images elicit similar activity as the transformed images, we should observe the same basic pattern repeat in blocks throughout all the transformations. For the pixel, V1 and DNN layer-2 representations, there is some degree of invariance (Fig. 6*F–H*), but the invariance was much stronger for the DNN conv-5 layer (Fig. 6*I*) and across IT neurons (Fig. 6*J*). While the low-level model representations were poorly matched to the IT representation, the match was quite high for the DNN conv-5 layer (*r* = 0.71, *p* < 0.00005). However, this match was still lower compared with reliability of the IT data (calculated as the corrected split-half reliability between dissimilarities obtained from two halves of the neurons, *r* = 0.92, *p* < 0.00005). Thus, the invariance observed in IT is not a trivial consequence of low-level visual representations but rather reflects non-trivial computations.

Taken together, our results show that the hierarchy of invariances in IT neurons is not trivially inherited from low-level visual representations, but rather reflects their underlying computational complexity, as revealed by a similar hierarchy in higher layers in deep neural networks.

## Discussion

Here, we have compared the dynamics of invariant object representations in IT neurons for four identity-preserving transformations: size, position, in-plane rotation, and in-depth rotations (view). Our main finding is that object representations in IT neurons evolve dynamically in time during the visual response: they generalize fastest across changes in size, followed by position and only later across rotations (both in-plane and in-depth). We obtained similar results using state-of-the-art deep convolutional neural networks, indicating that this ordering of invariances reflects their computational complexity. Below we discuss our findings in the context of the literature.

Our main finding is that, when invariances are compared after equating image changes, size and position invariance are stronger than rotation and view invariance. Although we have equated image changes across transformations using the net pixel change, it is possible that the representational change in the retinal or early visual cortical input to IT is not entirely balanced. However, we have shown that the ordering of invariances in low-level visual representations (pixels, V1, or initial layers of deep networks) is qualitatively different from that observed in IT. In the absence of more accurate models for retinal or V1 representations, our study represents an important first step in a balanced comparison of invariances. Our findings are consistent with the limited position and size invariance observed in midlevel visual areas like V4 (Pasupathy and Connor, 1999; Connor et al., 2007) and the increased invariance observed in IT (Rust and Dicarlo, 2010; Rust and DiCarlo, 2012). While IT neurons are well-known for their invariance to position, size, viewpoint, etc. (Tanaka, 1996; Connor et al., 2007; DiCarlo et al., 2012), to our knowledge, ours is the first study to directly compare the dynamics and magnitude of size, position, rotation, and view invariance in a balanced manner.

Our finding that view invariance develops later in time is consistent with the general consensus that it is a challenging problem since images undergo complex changes when objects are rotated in depth (Hayward and Tarr, 1997; Tarr et al., 1998; Biederman and Bar, 2000; Ratan Murty and Arun, 2015). Even in computational models, size and position invariance are seen in early layers and view invariance occurs only in later layers (Riesenhuber and Poggio, 1999; Wiskott, 2009; Leibo et al., 2015). However, even in these studies, image change was never equated in magnitude, and therefore, the finding that invariances are present in different layers could reflect the fact that some image changes were inadvertently smaller than others. Thus, our results could not have been anticipated given the existing literature on invariance in the ventral stream or from computational modeling.

Our finding that invariances differ in their time course may have interesting implications for behavior. If behavior is based directly on the early part of the visual response in IT neurons, it would predict that size or position invariance would be easier and faster than rotation and view. If behavior is based on later part of the visual response, then invariant behavior will be poor. We consider this unlikely, since invariance is extremely robust in behavior, and further, the low invariance late in time presumably only reflects the low neural response levels (Figs. 3*C*,*D* and 4*C*,*D*). We speculate that invariant representations may instead be maintained across time when they are task-relevant, either within IT or in downstream prefrontal regions. Finally, these differences between invariances may not even manifest in behavior. This could happen if objects used in the task are sufficiently dissimilar to be discriminated even using low-level visual representations (Foster and Gilson, 2002; Stankiewicz, 2002). Thus, finding parallel effects in behavior may require careful task design and calibration of objects.

The specific temporal ordering from size, position to rotation invariance in IT neurons could reflect some specific dynamic solution in the brain, or reflect the more general computational difficulty of these invariances. Our finding that rotation and view invariance are weaker even in deep convolutional neural networks supports the latter possibility, given that higher layers in deep neural networks match neural representations in high-level visual cortex (Khaligh-Razavi and Kriegeskorte, 2014; Yamins et al., 2014; Kheradpisheh et al., 2016). In sum, we conclude that object invariances in IT neurons develop in a specific temporal order reflective of their underlying computational complexity.

## Synthesis

Reviewing Editor: Tatyana Sharpee, The Salk Institute for Biological Studies Decisions are customarily a result of the Reviewing Editor and the peer reviewers coming together and discussing their recommendations until a consensus is reached. When revisions are invited, a fact-based synthesis statement explaining their decision and outlining what is needed to prepare a revision will be listed below. The following reviewer(s) agreed to reveal their identity: Charles Connor, Chou Hung.

The manuscript presents a careful analysis of how invariance to position, size, and rotation evolves in time in monkey IT cortex. The main points to address in the revision are to (1) analyze whether there are differences across neurons or stimuli in terms of the degree of invariance, (2) discuss potential limitations that arise from defining the similarity in pixel space, and (3) compare neural responses to different stages of processing in the network.

Reviewer 1:

This is an important and carefully executed comparison of position, size, and rotation invariance in monkey IT cortex. Small and large transformations in these dimensions are equated in terms of pixel correlation. Invariance is measured by correlation of the pattern of responses to 10 stimuli between reference and transformed conditions. The most solid general finding is that position and size invariance develop earlier than rotation invariance. In addition, the authors state that position and size invariance is stronger than rotation invariance. This was true at least in the 80-110 ms period after stimulus onset. This period is still relatively early in perceptual processing, and the difference disappears afterwards, which could indicate that rotation invariance is merely delayed but potentially equivalent on normal perceptual timescales. (The authors should discuss this.) The strength conclusion also depends on accepting the equivalence between transformation steps based on pixel similarity. This could be questioned since pixel-level similarity is only truly relevant at the level of rods and cones. At subsequent stages similarities in contrast borders, orientations, spatial frequencies, and high-order properties are more relevant measures of similarity. The authors deserve credit for attempting to quantify an equivalence between transformation magnitudes in position, size, and rotation, but they should discuss the limitations. The timing conclusion is less subject to this caveat.

A general problem with the analyses presented here is that they obscure potential differences across stimuli and differences across neurons. The basic analysis for each neuron is correlation between reference and transformed responses across all 10 stimuli. This throws out any potential information about strong invariance for specific stimuli. In particular, it might be imagined that a given neuron is strongly selective and invariant for one stimulus, but this quality is swamped in the initial analysis step by weaker, more variable responses to the other 9 objects. Thus, this step and all subsequent neural analyses based on it make the assumption that the same level of invariance holds for all objects, and that assumption is not necessarily valid.

The next analysis step is to average these correlation values across the neural population. This obscures the population distribution of invariance properties. A population average correlation of 0.3 could mean some neurons are highly invariant while others produce widely different responses. (Figure 5A suggests this, although it only shows invariance for small transformation steps; the larger transformation is of greater interest.) Or it could mean that all neurons show some limited degree of invariance. Readers will be interested to know whether there is a subpopulation of highly invariant IT neurons.

The high performance in the linear classifier analysis indirectly suggests that there is substantial information obscured by these analyses. I would like to know more about the subpopulation that supports the linear classifier results. How large is it? Is it anatomically clustered? Does it comprise neurons with sparse, invariant tuning for single stimuli? Or are the same neurons for high performance on multiple stimuli?

The authors need to analyze their data in ways that break out differences between stimuli and between neurons. They also need to analyze how performance of the linear classifier depends on stimuli (are some easier than others?) and on neurons (are there not groups of neurons tuned for specific stimuli and responsible for invariant recognition?).

SPECIFIC POINTS

“Thus, invariant neurons tend to be invariant along many identity-preserving transformations. This is concordant with the recent observation that selective IT neurons are selective along many dimensions” This sounds wrong to me. Invariance is exactly the opposite of selectivity.

(Simonyan & Zisserman 2015). We selected the layer in the neural network whose dissimilarities across all stimuli matched best with the IT data (conv5 layer; correlation with IT dissimilarities across all stimuli:

204 r = 0.78, p < 0.00005). This high r seems like a very important and revealing comparison of IT to a neural network, so it begs for more description. How was the network trained? Was it trained on the stimuli in this experiment? Is this correlation with dissimilarities in responses to the full stimulus set described in this paper? The average invariances are much higher than in IT. Why?

Line 77 typo; remove “and'. Otherwise very well written.”

Reviewer 2

Main comments:

1) It is unclear how the balancing was done at the pixel level. Were they based on units of pixel gun level? Silhouette? Candela? What was the background? Did the authors also test balancing based on model retina or V1 units?

2) The relative changes in position (0.9, 2.7 deg for 5 deg object) and size (0.87x, 0.71x) were much smaller than in previous reports. This direction of change in the stimulus parameters means that the results are not so surprising.

3) Fig. 3D and 4D. To what extent is the longer latency for rotations due to the neurons being worse for view-invariant classification for these objects (some of which were unnatural)? If you analyzed (e.g. train the classifier) on a subset of neurons that respond/perform equally well across size and view transformations, do you still see the latency difference? How about for other pairwise comparisons? Please discuss in text, e.g. after line 183, or as a separate section after line 195.

4) The model results for earlier layers should also be reported as a comparison. The expectation is that the poorer invariance for rotation and view should not already be present in the first layer. If it is already present in the first layer, it weakens the comparability of neural to model results. Either way, the comparison is interesting and can attest to the extent to which neural and model results coincide, and more interesting, where the gaps are in the deep learning approach to recognition.

Minor comments:

1) Fig. 4A. Boundaries for the linear classifier should be rotated and translated to look more like an equilateral triangle minus the corners. The classifiers are trained to pick out a single category, so each category should only have one hyperplane, not two.

2) Legends for Fig. 3B, Fig. 4. Spell out what *** and **** mean, not 'etc'.

3) The test was based on 10 objects, but aside from the pictures of 6 objects in Fig. 2, no description is given of the other 4 objects. In its current state, what comes across is that the study is based on coarse-level discrimination across a few objects in a few categories (4-legged animals vs transportation) that may not generalize to other categories or to fine-scale discriminations within categories. The authors may want to expand the description in the Stimuli section and discuss the limits of interpretation in the Discussion.
